# biomArker-guided Duration of Antibiotic treatment in hospitalised Patients with suspecTed Sepsis (ADAPT-Sepsis): A protocol for a multicentre randomised controlled trial

**DOI:** 10.1177/17511437231169193

**Published:** 2023-04-25

**Authors:** Paul Dark, Gavin D Perkins, Ronan McMullan, Danny McAuley, Anthony C Gordon, Jonathan Clayton, Dipesh Mistry, Keith Young, Scott Regan, Nicola McGowan, Matt Stevenson, Simon Gates, Gordon L Carlson, Tim Walsh, Nazir I Lone, Paul R Mouncey, Mervyn Singer, Peter Wilson, Tim Felton, Kay Marshall, Anower M. Hossain, Ranjit Lall

**Affiliations:** 1 Division of Immunology, Immunity to Infection and Respiratory Medicine, University of Manchester, Critical Care Unit, Northern Care Alliance NHS Foundation Trust, Salford Care Organisation, Greater Manchester, UK; 2 Warwick Medical School, Clinical Trials Unit, University of Warwick, Coventry, UK; 3 Wellcome Wolfson Institute for Experimental Medicine, School of Medicine, Dentistry and Biomedical Sciences, Queens University Belfast, Belfast, UK; 4 Anaesthetics, Pain Medicine and Intensive Care, Department of Surgery and Cancer, Faculty of Medicine, Imperial College, London, UK; 5 Clinical Biochemistry Department, Lancashire Teaching Hospitals NHS Foundation Trust, Sharoe Green Lane, Fulwood, Preston Lancashire, UK; 6 School of Health and Related Research, The University of Sheffield Western Bank, Sheffield, UK; 7 Cancer Research Clinical Trials Unit, Institute of Cancer and Genomic Sciences, University of Birmingham, Edgbaston, Birmingham, UK; 8 National Intestinal Failure Centre, Northern Care Alliance NHS Foundation Trust, Salford Care Organisation, Greater Manchester, UK; 9 Anaesthesia, Critical Care and Pain Medicine, Usher Institute, College of Medicine and Veterinary Medicine, University of Edinburgh, Edinburgh Royal Infirmary, Edinburgh, UK; 10 Clinical Trials Unit, Intensive Care National Audit and Research Centre, Napier House, London, UK; 11 Centre for Intensive Care Medicine, Experimental and Translational Medicine, Division of Medicine, Faculty of Medical Sciences, University College London, London, UK; 12 Clinical Microbiology, University College London Hospitals NHS Foundation Trust, London, UK; 13 Respiratory Academic Group, Division of Immunology, Immunity to Infection and Respiratory Medicine, University of Manchester, Wythenshawe Hospital, Manchester, UK; 14 Pharmacy and Pharmaceutical Sciences, School of Health Sciences, University of Manchester, Manchester, UK

**Keywords:** Antibiotic duration, sepsis, procalcitonin, C-reactive protein, biomarkers

## Abstract

**Aim:**

To describe the protocol for a multi-centre randomised controlled trial to determine whether treatment protocols monitoring daily CRP (C-reactive protein) or PCT (procalcitonin) safely allow a reduction in duration of antibiotic therapy in hospitalised adult patients with sepsis.

**Design:**

Multicentre three-arm randomised controlled trial.

**Setting:**

UK NHS hospitals.

**Target population:**

Hospitalised critically ill adults who have been commenced on intravenous antibiotics for sepsis.

**Health technology:**

Three protocols for guiding antibiotic discontinuation will be compared: (a) standard care; (b) standard care + daily CRP monitoring; (c) standard care + daily PCT monitoring. Standard care will be based on routine sepsis management and antibiotic stewardship. Measurement of outcomes and costs. Outcomes will be assessed to 28 days. The primary outcomes are total duration of antibiotics and safety outcome of all-cause mortality. Secondary outcomes include: escalation of care/re-admission; infection re-lapse/recurrence; antibiotic dose; length and level of critical care stay and length of hospital stay. Ninety-day all-cause mortality rates will also be collected. An assessment of cost effectiveness will be performed.

**Conclusion:**

In the setting of routine NHS care, if this trial finds that a treatment protocol based on monitoring CRP or PCT safely allows a reduction in duration of antibiotic therapy, and is cost effective, then this has the potential to change clinical practice for critically ill patients with sepsis. Moreover, if a biomarker-guided protocol is not found to be effective, then it will be important to avoid its use in sepsis and prevent ineffective technology becoming widely adopted in clinical practice.

## Background and rationale

Early, appropriate antimicrobial treatment for infection is a crucial part of emergency interventions aimed at improving sepsis survivorship.^1^ Choosing the right antimicrobial drugs and doses is crucial because inappropriate antibiotic therapy is associated with two-to-fourfold increase in risk of death under these circumstances.^2^

Once commenced, the optimum duration of antibiotic treatment is less certain.^3^ Fixed duration antibiotic courses (up to 14 days in some circumstances) have been widely used in the NHS because clinical signs and microbiology culture tests are not sufficiently useful for monitoring treatment efficacy in order to guide the decision to stop the administration of antibiotics.^4^ Daily clinical review of treatment and patient progress, performed alongside microbiology results and advice, do provide opportunities to limit patient exposure to broad-spectrum antibiotics while tailoring effective therapy for a proven infection – the so-called ‘Start smart - then focus’ approach.^4,5^

Readily available circulating serum proteins such as C-reactive protein (CRP) and procalcitonin (PCT) – the most intensively researched biomarkers – are often raised in sepsis and usually fall in response to effective treatments.^6^ This provides a potential opportunity to personalise the duration of antibiotic therapy which could lead to reductions in population antibiotic usage, adverse effects for patients, improved healthcare resource utilisation and downstream benefits relating to antimicrobial resistance – an urgent priority. These biomarkers, however, are part of a complex inflammatory response triggered not only by infection but by other stimuli such as trauma and surgery. Thus, unnecessarily prolonged antibiotic treatment may be commenced if guided solely by raised levels of these biomarkers as part of antibiotic initiation/escalation protocols.^7^ Alternatively, biomarker-guided antibiotic discontinuation protocols for critically ill patients have been associated with shorter treatment durations in some healthcare systems internationally^1,8^ and reduced mortality,^9^ but studies are at high risk of bias^1,8,9^ and with uncertain relevance to NHS practice.^10^ There is thus a need for a multicentre randomised controlled UK trial to determine whether an antibiotic treatment protocol based on monitoring CRP or PCT might safely allow a reduction in the duration of antibiotic therapy in patients with sepsis.

## Objectives

### Primary objective

To determine whether treatment discontinuation protocols based on monitoring CRP or PCT in hospitalised adult patients with suspected sepsis reduces the duration of antibiotic therapy compared with standard care while maintaining treatment safety as measured by mortality 28 days after randomisation.

### Secondary objectives

To determine adherence to biomarker treatment protocols and their effects on antibiotic consumption, infection and antibiotic adverse events, critical care and hospital length of stay, acquisition cost of antibiotics, longer-term mortality and cost-effectiveness in the NHS setting.

## Methods

### Trial design

This is a multicentre prospective, individual patient randomised, 3-arm, controlled, intervention-concealed clinical and cost effectiveness trial. The trial is managed by the Warwick Clinical Trials Unit and sponsored by the University of Manchester. The funding is provided by the National Institute for Health Research (NIHR) following a commissioned call from the Health Technology Assessment programme (15/99/02). The NHS main contractor is the Northern Care Alliance NHS Foundation Trust. The trial is coordinated by a Trial Management Group (TMG) and independent oversight is provided by a Trial Steering Committee (TSC) and a Data Monitoring Committee (DMC). The trial has been designed and will be reported in line with the CONSORT (Consolidated *S*tandards *o*f *R*eporting *T*rials) statement.^11^ Trial conduct has been planned in full conformance with the principles of the Declaration of Helsinki and Good Clinical Practice.

### Study setting

The trial was planned to take place in at least 32 acute care NHS hospitals with adult critical care units. Hospitals must provide evidence that they are able to participate in critical care research, have access to the relevant patient population, have routine clinical biochemistry services able to provide, or establish, daily CE-marked PCT and CRP quality-assured laboratory assays.

### Participant inclusion criteria

- Hospitalised adult patients at least 18 years of age
- Up to 24 h of initiation of empiric intravenous antibiotic treatments for a suspicion of sepsis^12^
- Likely to remain hospitalised and receiving intravenous antibiotic treatment for at least the next 72 h; and
- Requirement for critical care.

A 24-h recruitment window from initiation of antibiotics for sepsis is required to determine baseline biomarker values to guide subsequent protocolised treatment duration advice.^6,10^

### Participant exclusion criteria

- Prolonged (greater than 21 days) antimicrobial therapy (e.g. for endocarditis, cerebral/hepatic abscess, tuberculosis, osteomyelitis);
- Severely immunocompromised not caused by sepsis (e.g. neutropenia less than 500 neutrophils/µl);
- Any patient given, or anticipated to receive an IL-6 receptor inhibitor drug (e.g. tocilizumab or sarilumab) during their acute hospital admission;
- All treatments for suspected sepsis likely to be stopped within 24 h of its initiation because of futility;
- Consent declined; or
- Previously enrolled into this trial.

Co-enrolment of study participants into other trials will be considered by the TMG using national guidance.^13^

### Trial protocol

**Figure 1.**
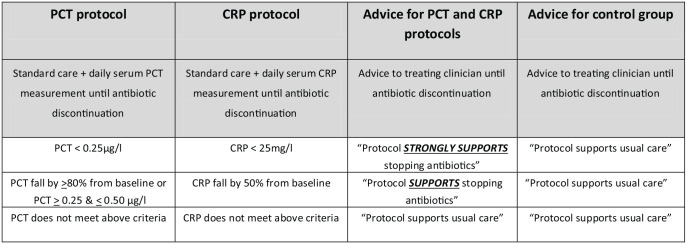
Trial biomarker-guided antibiotic discontinuation protocol.

Blood will be drawn daily in every trial patient participant from randomisation until discontinuation of antibiotics for the sepsis episode or discharge from hospital. The clinicians responsible for managing patients will receive daily standardised advice from the local research team on either standard care or on biomarker-guided antibiotic discontinuation. Advice will be based on daily serum testing of either (a) PCT or (b) CRP or (c) ‘no test’ (control group). The antibiotic discontinuation protocols are described alongside the standardised written advice for each group in Figure 1.

### Trial interventions

1. Procalcitonin arm. Standard care with daily serum PCT measurement until antibiotic discontinuation or hospital discharge. Daily feedback to clinical team based on PCT discontinuation protocol (Figure 1).
2. CRP arm. Standard care with daily serum CRP measurement until antibiotic discontinuation or hospital discharge. Daily feedback to clinical team based on CRP discontinuation protocol (Figure 1).

For the randomly allocated intervention arms, patient research blood collection (minimum of 2 ml research sample per day) and serum biomarker laboratory testing (PCT or CRP) will commence within the first 24 h following the initiation of intravenous antibiotics for sepsis. Daily research blood sampling, laboratory testing and subsequent advice in every patient will continue until antibiotics for the sepsis episode have been discontinued. Research blood sampling will not recommence if antibiotics are subsequently re-introduced within the 28-day study period following patient randomisation. If a participant is discharged from hospital on a course of antibiotics for the initial sepsis episode, the trial intervention will cease at the time of discharge. Phlebotomy and samples will be handled in line with agreed local standard care practice.

### Standard care

Daily blood sampling and delivery of sample to the laboratory. No biomarker testing of sample and feedback to clinical team will be based on usual care protocol (Figure 1).

Patients recruited to both control and intervention arms will receive standard NHS care for sepsis and antibiotic stewardship will follow Public Health England (PHE) guidance.^5^ Patients will be reviewed daily by their medical team with documented decisions on antibiotic treatment guided by standard clinical assessment and review of microbiological culture results. Routinely available laboratory data, such as white blood cell counts, will remain part of standard care for all patients recruited to our proposed trial because these are part of the current standard of NHS care for patients with sepsis.^5^ Daily clinical review of all patients with sepsis, as a standard-of-care, will allow incorporation of the intervention protocols for daily assessment of antibiotic discontinuation described in Figure 1.

### Outcome measures

#### Primary outcome measures

- Clinical effectiveness: Total duration (measured in days) of antibiotic treatment to 28 days following randomisation
- Safety: 28-day all-cause mortality following randomisation

#### Secondary outcome measures

Effectiveness and safety outcome measures to 28 days following randomisation:

- Antibiotic duration (measured in days) and dose (measured as Defined Daily Dose) for the sepsis episode
- Total antibiotic dose (measured as Defined Dailey Dose)
- Unscheduled care escalation/re-admission
- Infection relapse/recurrence requiring further antibiotic treatment
- Super-infection defined as new infection at a different anatomical site
- Suspected clinically relevant antibiotic adverse reactions
- Time to ‘fit’ for hospital discharge

All-cause mortality will be determined at 90 days

Health care system benefit outcomes

- Assessment of in-trial cost effectiveness (see below)
- Critical care unit length and level of stay^14^
- Hospital length of stay (days)

Safety reporting

- Adverse event data (see Supplemental Material)

#### Sample size estimate

A total sample size of 2760 will be required to detect both a mean of 1-day (0.93 days to be precise) reduction in antibiotic duration (using a mean antibiotic duration of 7 days, a pooled standard deviation of 6 days, 90% power, a significance level of 5%, with a 5% withdrawals rate) and a non-inferiority safety margin of 5.4% (using a 1-sided significance level of 2.5%, 90% power and 5% withdrawal rate) assuming 28-day mortality is 15% in both arms (see Supplemental Material).

### Randomisation, stratification and allocation concealment

Patient participants will be allocated at random to PCT, CRP and usual care groups in a 1:1:1 ratio using a computer-generated randomisation sequence produced by the minimisation method. Stratification factors will be (i) sepsis severity (sepsis or septic shock^12^), (ii) recruitment centre and (iii) surgery within the last 72 h or not. Allocation concealment will be provided by a centralised 24-h web-based randomisation system located at Warwick Clinical Trials Unit.

### Intervention concealment

Following patient recruitment, randomisation will be initiated by the local investigator team using a 24-h trial web-based system. Group assignment will be available to the laboratory service only through this web-based system and will be concealed from the patient and their relatives, the treating clinical teams and the local research staff. A research blood sample will be collected from each recruited patient, including standard care only (control group) and standard care plus biomarker-guidance (CRP and PCT intervention groups), to maintain group concealment. Research blood samples will be allocated a unique research study number and will be transported to the laboratory. The research number will not reveal the identity of the patient to laboratory staff. A sample will be collected and transported to the laboratory each day for every recruited patient (control and intervention groups) until antibiotics are discontinued by the clinical team responsible for patient care.

The trial website will be the route for routine reporting of research laboratory results and web-based automated advice will be generated for the clinical research teams daily for each patient. Automated, real-time, web-based, centre-specific, time-adaptive phasing factors will be used to maintain group concealment based on the speed of assays (CRP or PCT interventions) or no assay (control) for advice delivery. The clinical research team will deliver the daily written standardised advice to the clinicians responsible for patient care as described in Figure 1.

Study biomarker values will not be reported back to routine clinical service or patient care records.

### Protocol compliance

Screening; recruitment; reasons for exclusion and intervention adherence will be audited throughout the study by using data recorded in screening logs, Case Report Forms (CRFs) and during site visits. Intervention adherence will be captured using specific data recorded in the CRFs of adherence to biomarker-guided advice on antibiotic discontinuation – and reasons for non-adherence will be documented if it occurs.

A particular challenge for trials incorporating biomarker-guided antibiotic discontinuation protocols in sepsis is the variable but common use of CRP monitoring in this patient group, as identified by two independent national surveys of (a.) NHS hospitals and (b.) NHS clinical biochemistry service laboratories.^15^ These surveys indicated that CRP monitoring has not been used in the NHS as part of any defined antibiotic discontinuation protocols and there is considerable variation in CRP use in this setting. In addition, routine daily PCT measurement has not been widely adopted across the NHS to guide antibiotic duration decisions for sepsis, although the availability of PCT assays in the NHS has increased recently.^16^ Therefore, while we expect study centres to adopt a position of equipoise during the trial in terms of both protocolised CRP and PCT guided decisions on antibiotic duration, we accept that CRP may be measured outside of the study protocol if the treating clinician believes that this is an important part of a patient’s care. Any non-trial use of CRP in standard care that could impact on antibiotic treatment duration decisions will be recorded in the CRF and will be monitored at each site by the research team.

The non-trial use of PCT presents a particular challenge because it has the potential to influence antibiotic treatment duration decisions. For non-trial use, study centres with access to routine PCT are likely to use identical PCT-guided antibiotic stopping rules to those in the trial protocol and that are recognised internationally.^15^ Therefore, maintaining equipoise for the purposes of this trial involves avoiding PCT use in recruited patients. Any non-trial use of PCT to 28-days post randomisation will be reported as a protocol deviation and will be monitored at each site by the research team.

### Data collection and management

**Figure 2.**
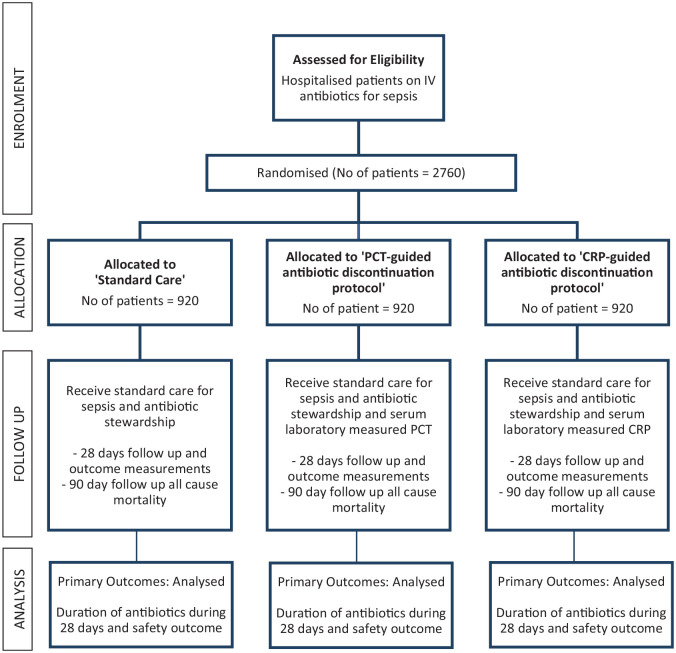
Trial flow diagram.

Data will be collected using a local paper CRF and web-based secure remote data capture system. Clinical data will be collected up to 28 days after randomisation as outlined in the study schematic (Figure 2) and detailed in Table 1. Participant identification in both the CRF and web-based system will be through a unique study number. Data will be collected daily from the time the patient is considered for entry into the trial through to their discharge from hospital. If a participant is transferred to another hospital, the site research team will liaise with the receiving hospital to ensure complete data collection. If a participant is discharged into the community prior to day 28, the site research team will access routine electronic healthcare records, family doctors or the patient and/or relative in order to complete collection of the day 28 follow up data.

**Table 1.** Schedule of delivery of trial interventions and data collection.

Visit day	1				2				3	4	5	6	7	8–28
Screening	✓													
Informed Consent (Patient consent/ Consultee /Guardian/Welfare Attorney/ Retrospective Patient Information & consent)	Patient / Consultee (Guardian/Welfare Attorney) Opinion/consent will be obtained initially. Retrospective patient consent will be obtained when/if the patient has recovered mental capacity during acute hospital care.													
Medical history and baseline characteristics	✓													
Inclusion/exclusion criteria	✓													
Randomisation	✓													
Baseline research blood sample	✓													
Intervention								Biomarker-guided antibiotic discontinuation						
SOFA score	✓								✓				✓	
Adverse events					✓				✓	✓	✓	✓	✓	✓
Follow-up														
Daily collection of clinical information, infection status, antibiotic use and care environment	✓				✓				✓	✓	✓	✓	✓	✓
Final visit	*If the patient is discharged to another hospital or to the community within 28 days following randomisation, the local research team will contact the patient and their treating health care professional (hospital physician or General Practitioner) to collect outstanding information about the stated primary and secondary outcomes.*													

All-cause mortality rates at 90 days will be collected using NHS Digital and the Intensive Care National Audit and Research Centre.

Patient data on disease severity will be collected using the Case Mix Programme (England, Northern Ireland and Wales) and its equivalent in Scotland (Scottish Intensive Care Society Audit Group). All-cause mortality rates at 90 days will be collected using NHS Digital and equivalents in Northern Ireland and Scotland.

### Statistical and health economic analysis plan (see Supplemental Material)

#### Regulatory and ethical approval

The Medicines and Healthcare products Regulatory Agency (MHRA) confirmed that their approval for the trial is not required because clinical decisions about antibiotic initiation and drug choice are not the object of this study and will be at the clinical judgement of the treating clinicians.

Ethical approval was from South Central – Oxford C Research Ethics Committee (REC) on 20th October 2017, Integrated Research Application System (IRAS) UK 209815 REC Ref: 17/SC/0434, and IRAS (Scotland) 234179, REC Ref: 17/SS/0125.

## Discussion

The ADAPT-Sepsis trial aims to determine whether a treatment protocol based on monitoring CRP or PCT safely allows a reduction in duration of antibiotic therapy in adult patients with sepsis and to assess cost effectiveness. This will be the largest randomised trial evaluating biomarker-guided antibiotic discontinuation in sepsis to date.^3^

The evidence-base for biomarker-guided antibiotic duration in sepsis was considered in a recent NIHR HTA commissioned systematic review.^8^ Based on this review, subsequent NICE guidance^10^ concluded that there is currently insufficient evidence to recommend routine adoption of PCT in the NHS. Furthermore, NICE recommended that research should be performed within the NHS for guiding decisions to stop antibiotic treatment in people with confirmed or highly suspected sepsis. NICE guidance also recognised that CRP is very likely to be monitored in the NHS during sepsis care, but there is a paucity of prospectively tested CRP-based algorithms and interventional data regarding efficacy and safety for guiding antibiotic duration.^10^ Therefore, the ADAPT-Sepsis trial has been designed to respond to these evidence gaps identified by NICE and has been funded by NIHR HTA following a specific commissioning brief call (15/99).

The health technologies being assessed are routinely available laboratory measured PCT and CRP-guided antibiotic discontinuation protocols aimed at safe reductions in antibiotic treatment duration. Antibiotic discontinuation protocol design was informed by the best available evidence. For PCT, a systematic review^8^ of intervention trials in sepsis revealed that PCT algorithms are based on multiple decision thresholds to guide antibiotic treatment in trial intervention arms, with final treatment decisions always remaining at the discretion of the treating clinician.^10^ Detailed PCT algorithms varied between studies; however, all discontinuation algorithms included a component that encouraged or strongly encouraged discontinuation of antibiotics when the PCT level was <0.25 µg/l and/or encouraged discontinuation of antibiotics when the PCT level was <0.5 µg/l. A fall in PCT from baseline by ⩾80% was incorporated into some algorithms to encourage antibiotic discontinuation.

To the best of our knowledge, there is only one randomised trial using CRP-guided antibiotic treatment discontinuation in sepsis.^17^ A CRP-based algorithm was compared with a PCT-guided algorithm in determining antibiotic duration – with both algorithms including antibiotic discontinuation rules based on relative declines and absolute biomarker thresholds. The CRP protocol had an absolute treatment discontinuation threshold at ⩽ 25 mg/l and relative discontinuation threshold when CRP fell by at least 50% from baseline. We could find no other prospectively tested CRP-based algorithm reporting interventional data regarding efficacy and safety for guiding antibiotic discontinuation.

The trial will adopt two threshold levels for both PCT and CRP antibiotic discontinuation guidance to embrace the best evidence identified by NICE and to align treatment advice that facilitates a biomarker intervention concealment strategy (Figure 1). Randomised controlled trials for biomarker-guided antibiotic treatment protocols for sepsis to date have been open-label and, therefore, at risk of bias (e.g. performance bias) – an acknowledged challenge.^1,8–10^ ADAPT-Sepsis is the first trial internationally to incorporate an intervention concealment strategy aimed at reducing the risk of bias, improving trial quality and delivering the best evidence for patient care.

In conclusion, if this trial finds that a treatment protocol based on monitoring CRP or PCT safely allows a reduction in duration of antibiotic therapy, and is cost effective, then this has the potential to change clinical practice in terms of how patients with sepsis are managed. Moreover, if a biomarker-guided protocol is not found to be effective, then it will be important to avoid its use in sepsis and prevent ineffective technologies becoming widely adopted in clinical practice.

## Supplemental Material

sj-docx-1-inc-10.1177_17511437231169193 – Supplemental material for biomArker-guided Duration of Antibiotic treatment in hospitalised Patients with suspecTed Sepsis (ADAPT-Sepsis): A protocol for a multicentre randomised controlled trial Click here for additional data file. Supplemental material, sj-docx-1-inc-10.1177_17511437231169193 for biomArker-guided Duration of Antibiotic treatment in hospitalised Patients with suspecTed Sepsis (ADAPT-Sepsis): A protocol for a multicentre randomised controlled trial by Paul Dark, Gavin D Perkins, Ronan McMullan, Danny McAuley, Anthony C Gordon, Jonathan Clayton, Dipesh Mistry, Keith Young, Scott Regan, Nicola McGowan, Matt Stevenson, Simon Gates, Gordon L Carlson, Tim Walsh, Nazir I Lone, Paul R Mouncey, Mervyn Singer, Peter Wilson, Tim Felton, Kay Marshall, Anower M. Hossain and Ranjit Lall in Journal of the Intensive Care Society

sj-docx-2-inc-10.1177_17511437231169193 – Supplemental material for biomArker-guided Duration of Antibiotic treatment in hospitalised Patients with suspecTed Sepsis (ADAPT-Sepsis): A protocol for a multicentre randomised controlled trial Click here for additional data file. Supplemental material, sj-docx-2-inc-10.1177_17511437231169193 for biomArker-guided Duration of Antibiotic treatment in hospitalised Patients with suspecTed Sepsis (ADAPT-Sepsis): A protocol for a multicentre randomised controlled trial by Paul Dark, Gavin D Perkins, Ronan McMullan, Danny McAuley, Anthony C Gordon, Jonathan Clayton, Dipesh Mistry, Keith Young, Scott Regan, Nicola McGowan, Matt Stevenson, Simon Gates, Gordon L Carlson, Tim Walsh, Nazir I Lone, Paul R Mouncey, Mervyn Singer, Peter Wilson, Tim Felton, Kay Marshall, Anower M. Hossain and Ranjit Lall in Journal of the Intensive Care Society
