# Supplementary material for: biomArker-guided Duration of Antibiotic treatment in hospitalised Patients with suspecTed Sepsis (ADAPT-Sepsis): A protocol for a multicentre randomised controlled trial
Source: J Intensive Care Soc. 2023 Apr 25;24(4):427–34. doi: 10.1177/17511437231169193 (PMC10572477; doi:10.1177/17511437231169193)
Supplement: sj-docx-1-inc-10.1177_17511437231169193 – Supplemental material for biomArker-guided Duration of Antibiotic treatment in hospitalised Patients with suspecTed Sepsis (ADAPT-Sepsis): A protocol for a multicentre randomised controlled trial [file sj-docx-1-inc-10.1177_17511437231169193.docx]

**Supplementary materials (methods)**

**1. Sample size estimate**

A total sample size of 2760 will be required to detect both a mean of 1-day (0.93 days to be precise) reduction in antibiotic duration (using a mean antibiotic duration of 7 days, a pooled standard deviation of 6 days, 90% power, a significance level of 5%, with a 5% withdrawals rate) and a non-inferiority safety margin of 5.4% (using a 1-sided significance level of 2.5%, 90% power and 5% withdrawal rate) assuming 28-day mortality is 15% in both arms.

The minimally important clinical difference of 1-day reduction of antibiotic use was informed by a systematic review^1^ of biomarker-guided antibiotic treatment duration for sepsis illustrating a mean difference between standard care and PCT biomarker-guided therapy of 1.2 (95%CI: 1.07, 1.33) days and a mean difference of 3.85 (95%CI: 0.92, 6.78) days when including a broader range of studies in critically ill patients at high risk of sepsis. There are estimated to be at least 200,000 cases of sepsis/annum across the UK^2^. Therefore, with an average antibiotic saving of at least 1.2 days for each patient with sepsis across the whole NHS (likely involving broad-spectrum antimicrobial agents) this could produce a meaningful change in practice in terms of antibiotic exposure which would also be cost-saving and more effective^2^.

The estimate for antibiotic treatment duration for standard sepsis care was derived from a number of sources. Consensus international management guidelines have recommended 7-10 days duration^3^ and UK guidance of 7 days from Public Health England^4^. In addition, Jong and colleagues^5^ derived a standard deviation of 6-day duration of antibiotic use from six relevant randomised controlled trials and prospective studies with a mean treatment duration of 8-days.

All-cause mortality in standard care was informed by a systematic review of randomised controlled trials of biomarker-guided antibiotic duration in sepsis^1^, ranging from 12.5% - 24.0%, primarily in critical care unit settings. Following the implementation of international management guidelines for sepsis, there is observational evidence to suggest that mortality rates are declining^6^. For this reason, we estimated the mortality rate for sepsis in the standard care arm will be 15%.

The trial has been designed to meet recommendations for absolute non-inferiority margins of <7% with respect to all-cause mortality (safety) for clinical trials that assess antibiotic drug efficacy in critically ill patients^7^. ADAPT-Sepsis, therefore, has been designed to help provide improved safety evidence when compared with the largest PCT interventional studies to date, Jong^5^ and Bouadma^8^, who set their absolute non-inferiority mortality margins at 8% and 10% respectively.

**2. Adverse outcomes reporting**

Any untoward medical occurrence in a patient participant, which does not necessarily have a causal relationship with the research, is defined as an Adverse Event (AE) and will be recorded on the patient’s Case Report Form (CRF). AEs will be graded according to severity, seriousness, causality and expectedness. All those AEs meeting the definition as serious (SAEs) will be submitted to the Warwick Clinical Trials Unit within 24 h of the investigator becoming aware.

Any clinically relevant antibiotic related reactions occurring from the time of randomisation until 28 days will be recorded as pre-defined trial outcomes. Events will be deemed clinically relevant and related if antibiotic treatment has been changed due to a suspected reaction to the previous antibiotic. The following clinically relevant antibiotic related events will be recorded in the CRF and do not need separate recording as AEs:

- Anaphylaxis
- Gastrointestinal
- Haematological
- Hepatobiliary
- Renal
- Neurological
- Dermatological
- Cardiac
- Muscular
- Clostridium Difficile diarrhoeal infection
- Multi-drug resistant organism
- Other (clinician’s discretion)

The primary events of infection and sepsis are classified as pre-existing conditions in the trial. As such, the occurrence or expected progression of infection and sepsis-related events, including death, will occur. These events will be recorded in the medical records and assessed in the same way as other AEs. However, they are not usually recorded as AEs, unless they are thought to be related to participant involvement in the trial. Specifically, clinical outcomes from infection and sepsis, and outcomes related to the interventions of biomarker-guided decision on antibiotic duration, including clinically relevant antibiotic related reactions listed above, are exempt from adverse event reporting. The following events will be collected as outcomes and will be recorded in the relevant CRF so do not need to be reported as SAEs:

- All-cause mortality at 28 and 90-days post randomisation (unless the site investigator feels the death is related to the participation in the trial)
- Cardiovascular failure, including the need for vasopressors / inotropes
- Respiratory failure, including mechanical ventilation and acute lung injury
- Hepatic failure
- Renal failure, including the need for renal replacement therapy
- Haematological / coagulation failure, including thrombocytopaenia
- Neurological failure
- Unscheduled care escalation/re-admission
- Infection relapse/recurrence requiring further antibiotic treatment
- Super-infection defined as new infection at a different anatomical site
- Suspected antibiotic adverse reactions

Clinical decisions about antibiotic initiation and drug choice are not the object of this trial and will be at the clinical judgement of the treating clinicians in each of the three study groups.

**References**

1. Westwood ME, Ramaekers BLT, Whiting P, et al. Procalcitonin testing to guide antibiotic therapy for the treatment of sepsis in intensive care settings and for suspected bacterial infection in emergency department settings: a systematic review and cost-effectiveness analysis. *Health Technol Assess* 2015: 19(96).

2. National Institute of Health and Care Excellence. Procalcitonin testing for diagnosing and monitoring sepsis (ADVIA Centaur BRAHMS PCT assay, BRAHMS PCT Sensitive Kryptor assay, Elecsys BRAHMS PCT assay, LIAISON BRAHMS PCT assay and VIDAS BRAHMS PCT assay) (DG18). NICE Guidance, October 2015.

3. Rhodes A, Evans LE, Alhazzani W et al. Surviving Sepsis Campaign: International Guidelines for Management of Sepsis and Septic Shock: 2016. *Intensive Care Med.* 2017; 43: 304-377.

4. Public Health England. Start Smart - Then Focus: Antimicrobial Stewardship Toolkit for English Hospitals. PHE Publications Gateway number: 2014828, March 2015.

5. de Jong E van Oers JA Beishuizen A, et al. Efficacy and safety of procalcitonin guidance in reducing the duration of antibiotic treatment in critically ill patients: a randomised, controlled, open-label trial. *Lancet Infect Dis.* 2016; 16: 819-827.

6. Levy MM, Rhodes A, Phillips GS, et al. Surviving Sepsis Campaign: association between performance metrics and outcomes in a 7.5-year study. *Intensive Care Med.* 2014; 40:1623–33.

7. Sorbello A, Komo S and Valappil T. Noninferiority margin for clinical trials of antibacterial drugs for nosocomial pneumonia. *Drug Information Journal* 2010; 44:165-76.

8. Bouadma L, Luyt C-E, Tubach F, et al. Use of procalcitonin to reduce patients’ exposure to antibiotics in intensive care units (PRORATA trial): a multicentre randomised controlled trial. *Lancet* 2010; 375:463–74.
