# Supplementary material for: biomArker-guided Duration of Antibiotic treatment in hospitalised Patients with suspecTed Sepsis (ADAPT-Sepsis): A protocol for a multicentre randomised controlled trial
Source: J Intensive Care Soc. 2023 Apr 25;24(4):427–34. doi: 10.1177/17511437231169193 (PMC10572477; doi:10.1177/17511437231169193)
Supplement: sj-docx-2-inc-10.1177_17511437231169193 – Supplemental material for biomArker-guided Duration of Antibiotic treatment in hospitalised Patients with suspecTed Sepsis (ADAPT-Sepsis): A protocol for a multicentre randomised controlled trial [file sj-docx-2-inc-10.1177_17511437231169193.docx]

**KEY DOCUMENT REVIEW/APPROVAL**


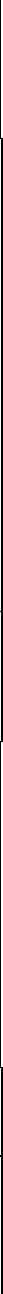


| **Trial:** | Biom**A**rker-guided **D**uration of **A**ntibiotic treatment in hospitalised **P**a**T**ients with suspected **Sepsis**: the **ADAPT-Sepsis** Trial. | | |
| --- | --- | --- | --- |
| **Chief Investigator:** | **Professor Paul Dark** | | |
| **Document:**  D Protocol / amendment*  D PIS/ amendment*  D Consent form / amendment*  D CRF / amendment*  D Publication(s) *(add details)*  D Risk Assessment  D Monitoring Plan  D Statistical Analysis Plan  D Economic Evaluation Analysis Plan  D Data Management Plan  * *excluding minor amendments e.g. typo errors, formatting* | | **Version: 1.1** | **Date: 08-03-2022** |
| **Reviewers:**  By signing this form, you confirm the content of the document specified has been reviewed and found to be correct. | | | |
| **Name** | **Role in trial** | **Signature** | **Date** |
| Dr Anower Hossain | *Statistician* | 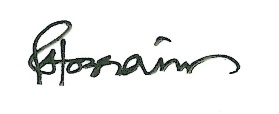 | 08/03/2022 |
| Professor Ranjit Lall | *Senior Statistician* | 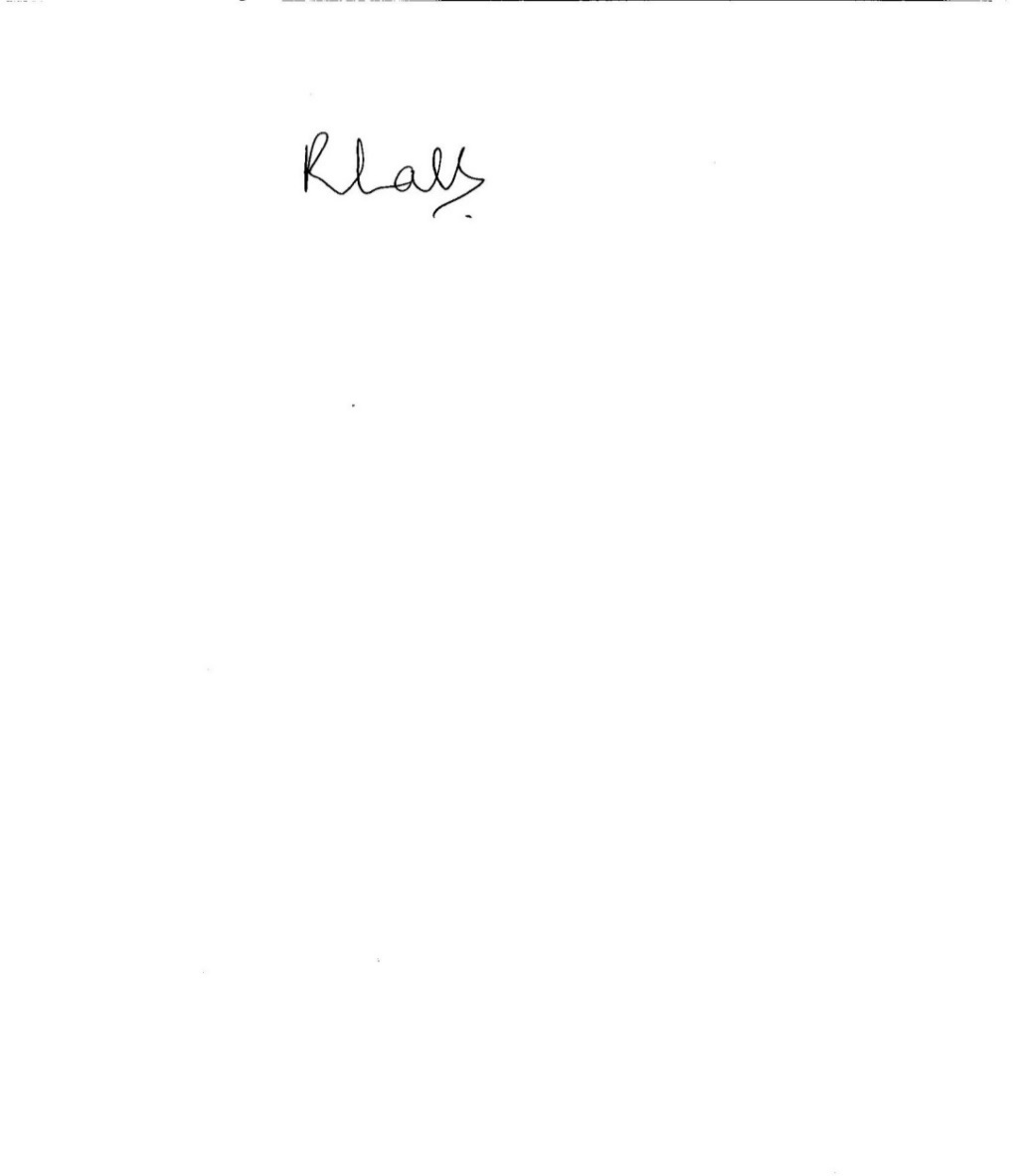 | 8/03/2022 |
| Dr Ly-Mee Yu | *DMC chair* | 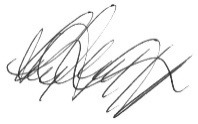 | 23 March 2022 |
| **Chief Investigator Approval:**  I confirm that the document specified has been reviewed and I approve the use of this version. | | | |
| Signature:  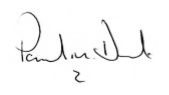 | | Date: 8^th^ March 2022 | |

*The signed form should be filed with the approved document in the TMF.*

Key Document Review/Approval Form
Version 3.0: 4 February 2018

**Introduction**

*Background and rationale*

Sepsis is a common life-threatening condition where early antibiotic treatment is an important factor in determining survival^1^. For patients with suspected sepsis, it is recommended that antibiotics are commenced within 1 hour, an NHS standard incentivised by a payment framework^2^. However, once commenced, the duration of antibiotic treatment is less certain, as identified within the brief for this call. Fixed duration antibiotic courses are widely used in the NHS because clinical signs and microbiology culture tests are not useful to monitor treatment efficacy to guide the decision to stop antibiotics^3^. Readily available circulating serum proteins such as C-reactive protein (CRP) and procalcitonin (PCT) are often raised in sepsis and usually fall in response to effective antibiotic treatment^4^. This provides a potential opportunity to personalise antibiotic duration which could lead to reductions in population antibiotic burden, adverse effects for patients, improved healthcare resource utilisation and downstream effects relating to antimicrobial resistance - an urgent priority. Biomarker-guided antibiotic discontinuation protocols in sepsis have been associated with shorter treatment durations in other healthcare systems internationally^5, 6^, but according to NICE, studies are low quality and with uncertain relevance to NHS practice.

The ADAPT-Sepsis trial is a randomised controlled trial to determine the clinical and cost-effectiveness of both CRP and PCT-guided antibiotic duration, using discontinuation protocols, when compared with standard practice in hospitalised adults with sepsis. A more detailed explanation of the background, rationale, and intervention and trial design can be found in the protocol paper. A brief overview of the trial is presented in this paper.

This paper presents the statistical analysis plan for the ADAPT-Sepsis trial which has been developed in line with published guidelines and the planned cost-effectiveness analysis.

*Objectives*

The primary objective of this trial is to determine whether treatment protocols based on monitoring CRP or PCT in hospitalised adult patients with suspected sepsis reduces the duration of antibiotic therapy (superiority) while maintaining treatment safety (non-inferiority) as measured by 28-day mortality. A supplementary objective is to estimate the relative cost-effectiveness of each protocol.

**Study methods**

*Trial design*

The ADAPT-Sepsis is a multi-centre three-arm randomised controlled trial with internal pilot. Participants are randomly allocated using a ratio 1:1:1 to CRP: PCT: Usual care. A computerised minimisation randomisation system was used, created by the Warwick Clinical Trials unit, to randomise participants. The randomisation allocation was made following consent processes and baseline assessments. Stratification factors were (i) sepsis severity (sepsis or septic shock), (ii) recruitment centre and (iii) surgery within the last 72 hours or not.

*Primary and secondary outcomes*

The primary outcomes are effectiveness outcome of total duration of antibiotics used to 28 days post randomisation and safety outcome of 28 days all-cause mortality. Secondary outcomes include escalation of care/re-admission; infection re-lapse/recurrence; dose of antibiotics; length and level of critical care stay, length of hospital stay, and 90-days all-cause mortality.

*Sample size*

A total sample size of 2760 would be able to detect both a mean of 1-day reduction in antibiotic use duration (using a mean antibiotic duration of 7 days, a pooled standard deviation of 6 days, 90% power, a significance level of 5%, with a 5% withdrawals rate) and a non-inferiority safety margin of 5.4 % (using a 1-sided significance level of 2.5%, 90% power and 5% withdrawal rate) assuming 28-day mortality is 15%.

*Timing of final analysis*

The end of the trial is defined when the last recruited participant completed 28-days follow-up post randomisation. Once all the data have been entered onto the database, the data will be fully validated and cleaned after which the database will be locked, and the final analyses will be undertaken.

*Timing of outcome assessments*

Primary and secondary outcome data will be collected daily from randomisation until hospital discharge. Data will also be collected post hospital discharge up to 28-days post randomisation. All-cause mortality will be collected at 90 days using linked data. Any SAE’s will also be reported up to 28 days post-randomisation.

**Statistical principles**

*Confidence intervals and p-values*

All statistical tests will be two-sided at the 5% significance level except for the non-inferiority safety test that will use a 1-sided significance level of 2.5%^7^. The estimate, 95% confidence interval (95% CI) and P value will be reported for each test undertaken.

*Analysis populations*

All analyses will be based on ‘Intention-to-treat’ (ITT) following ICH E9 guidelines. The participants will be analysed according to the treatment they were randomised to, irrespective of the treatment they actually received. All participants will be included in the analysis, regardless of whether they adhered to the protocol.

The Per Protocol (PP) population is usually defined as all participants who completing the study without major protocol deviations. We will identify the participants in this study who completed the study without major protocol deviations and then perform a PP analysis as secondary analysis.

*Adherence and protocol deviations*

The idea of site protocol adherence has been developed over a series of TMG discussions. Protocol processes have been developed through 4 key steps to help identify ‘protocol adherence’ and will be closely monitored during the trial. These include assessment of blood samples, production of advice, delivery of advice and the decisions taken for antibiotic treatment.

**Trial population**

*Screening data*

A detailed summary of the screening data will be presented as frequencies and percentages to describe the representativeness of the trial sample. This will include total number of hospitalised adult patients screened for eligibility, and number (percentage) of patients randomised to intervention arms.

*Eligibility*

The ADAPT-Sepsis trial protocol provides full details of the eligibility criteria (inclusion/exclusion criteria). The eligibility will be summarised using frequencies and percentages to describe how many patients were:

- Eligible and randomised
- Eligible and not randomised (summarising the main reasons for not randomised)
- Ineligible and not randomised (summarising the main reasons for exclusion)

*Recruitment*

A Patient Flow diagram will be used to illustrate the flow of participants throughout the trial. This will include:

- Number of patients screened for eligibility

- Number of eligible patients randomised to each intervention arm

- Number of withdrawals, died and lost to follow-up after randomisation

- Number of randomised patients included in the final analyses at the primary endpoint(s)

*Withdrawal and follow-up*

Participants may be discontinued from the trial at any time. They can either withdraw from the intervention alone but remain on follow-up or they can withdraw completely from the trial. Unless a participant explicitly withdraws their consent, they will be followed-up wherever possible and data collected as per the protocol until the end of the trial. The number of withdrawals from the trial will be summarised by treatment arms, that is, how many participants withdrew from intervention alone but remained on follow-up and how many withdrew completely from the trial. In addition, the withdrawal timings will also be summarised by treatment arms. It will include withdrawals after randomisation but before hospital discharge, and withdrawals after hospital discharge but before 28 days follow-up. The withdrawal decision i.e. decision made by the participant, consultant, clinical team, antibiotic stopped due to futility, required >21 days of antibiotics post randomisation and other, will be summarised by treatment arms. Follow-up rates will be computed at the 28-day and 90-day follow-up time-points. At 90 days, only all-cause mortality data will be collected using NHS digital and the Intensive Care National Audit and Research Centre.

*Baseline patient characteristics*

The baseline demographic characteristics and pre-randomisation clinical measures of all randomised participants will be summarised by treatment arms. Moreover, important process data (e.g. date and time of hospital admission and ICU/HDU admission) will also be collected and summarised by treatment arms. Continuous data will be summarised using the statistics the number of participants (n), mean, standard deviation (sd), median and interquartile range (IQR) whereas categorical data will be summarised using the statistic the number (%) of participants. The table below lists the demographic, clinical measures and process variable data that will be collected and summarised.

- Age
- Gender
- Suspected septic shock
- Surgery within last 72 hours
- Core body temperature
- White cell count
- Critical care admission category
- Critical care origin
- Initial care bundle
- qSOFA score
- SOFA score
- APACHE II score
- Infection data:
  1. - Community/hospital acquired infection
  2. - Site of infection
  3. - Causative microorganism identified for infection
  4. - If yes to the above, detail pathogen
- Process of care/adherence variables:

Time from:

- Hospital admission to ICU/HDU admission
- Hospital admission to first diagnosis of suspected sepsis or suspected septic shock
- Randomisation to first research blood sample taken
- First blood sample taken to first blood sample delivered to laboratory

**Analysis**

*Outcome definitions*

The primary outcomes of the trial are (i) total duration of antibiotic treatment to 28 days following randomisation (superiority) measured in days as primary clinical effectiveness outcome, and (ii) 28-day all-cause mortality (non-inferiority) following randomisation as primary safety outcome. For the clinical effectiveness outcome, the use of antibiotics data will be recorded to the point where the participant stopped antibiotics. The use of antibiotics will also be recorded if participants either restarts antibiotics in hospital or discharged from hospital and the re-admitted and receives systemic antibiotics or discharged with antibiotics provided that these events are within 28 days following randomisation. The duration of antibiotics use will be calculated for each participant as the sum of the duration of each course of antibiotics administered from randomisation to day 28. For the safety outcome, all-cause mortality status on day 28 following randomisation will be recorded.

The Secondary effectiveness and safety outcome measures to 28 days following randomisation are

- Antibiotic duration and dose for the initial sepsis episode (duration in days from randomisation and dose measured as Defined Daily Dose)
- Antibiotic dose (measured as Defined Daily Dose)
- Unscheduled care escalation/re-admission
- Infection relapse/recurrence requiring further antibiotic treatment
- Super-infection defined as new infection at a different anatomical site
- Suspected antibiotic adverse reactions
- Time to ‘fit’ for hospital discharge

The 90-days all-cause mortality will also be analysed as secondary outcome using linked data, for example, from NHS Digital and the Intensive Care National Audit and Research Centre.

*Analysis methods*

Participants’ baseline characteristics and outcomes will be summarised mean and standard deviation if data are continuous or frequency and percentage if data are categorical. The median and interquartile range will be presented if data are non-normal.

The primary effectiveness analyses will assess whether any of the biomarker interventions are better than standard care. The primary analysis approach for the duration of antibiotics will be intention-to-treat (ITT). ITT analysis will consist of all randomised patients and will assess the effect of treatment assignment. For the ITT analysis, linear mixed effects regression models will be fitted to estimate the treatment effect having adjusted for age, gender and the stratification variables (sepsis severity, recruiting centre (random effect) and surgery within the last 72 hours). After fitting the model, we will investigate the validity of the assumption of normality. Alternative methods of analyses will be considered if the distribution of the primary effectiveness outcome is non-normal. In this trial, participants may die before antibiotic therapy is discontinued. In such cases the outcome is undefined and is referred to as ‘truncated by death’. A crude comparison between the survivors on each treatment arm may give rise to biased outcome comparisons. Therefore, we will consider undertaking some sensitivity analyses to estimate treatment effect having account for the deaths. This will explore different approaches to analysis of ICU trials, that allow incorporation of multiple relevant outcomes, including death, into a single overall measure (e.g., the win ratio^8^, global ranking score^9^, and weighted composite outcome^10^.

The primary safety outcome (28-day all-cause mortality) is based on assessing non-inferiority and we specify the null hypothesis that the biomarker interventions are much worse compared with standard-of-care. The primary safety outcome will be analysed using mixed effect logistic regression models, using the same adjustments variables as the primary effectiveness analysis. We will derive risk difference from the fitted logistic regression model to compare the non-inferiority margin for the safety outcome. The unadjusted and adjusted estimates and the 95% confidence intervals will be compared with the non-inferiority margin to accept/reject the null hypothesis. Therefore, our primary effectiveness outcome (antibiotic duration) will not provide all the information as regards the treatment effect. Safety (28-day all-cause mortality) is paramount - only if an intervention arm is not worse than the control (standard care only) will it be acceptable.

Secondary outcomes will be assessed using an ITT approach. Continuous secondary outcomes will be analysed in the same way to the primary outcome and the categorical outcomes will be assessed using mixed effects logistic regression models. Time-to-event outcomes will be analysed using Cox proportional hazards models. For all analyses, the adjusted effect estimates will be presented along with their associated 95% confidence interval (CI). In addition, the primary outcomes will also be analysed using Bayesian method as secondary analysis considering both non-informative and informative priors.

Exploratory sub-group analyses for primary effectiveness outcome will be reported using 99% confidence intervals. Linear mixed effect regression will be used with interaction terms (treatment group by sub-group) to estimate sub-group effects. The following sub-groups selected from a recent report about the management of infection causing sepsis in the NHS will be explored:

- - Community-acquired pneumonia (CAP)
  - Hospital-acquired pneumonia (HAP)
  - Urinary tract infection (UTI)
  - Intra-abdominal infection
  - Infection with positive blood culture

In addition, community acquired, hospital acquired infections and SARS-COV-2 will be analysed as subgroup. Moreover, we will do the following subgroups analysis

- - Sepsis vs Septic Shock
  - Ward vs ICU where the intervention was stopped
  - Surgery vs non-surgery on previous 72 hours

An exploratory analysis will be done to investigate weekend effect on the primary effectiveness outcome, the total duration of antibiotic treatment to 28 days from randomisation. Monitoring of the protocol processes and compliance measures is important in this trial (as detailed in section 4). These measures will be monitored and reported to the DMC throughout the trial. A final summary of the protocol processes and compliance will be presented at the end of the trial.

**Handling missing data**

Every effort will be made to minimise missing data both in baseline and outcome in the trial. Missing data will be assessed carefully. For the primary outcome (duration of antibiotics), start and stop dates for each course of antibiotics are required in order to compute the total duration for each participant. There will be no missing for start dates as the start dates and times will be the randomisation dates and times. In the event that stop dates are unobtainable and thus missing, we will first look to see if the participant either withdrew completely or died. If so, we will use the withdrawal/death date as the stop date to estimate the duration for a particular course of antibiotics. If they did not withdraw or die, then we would impute the duration for that particular course of antibiotics using the mean duration of all antibiotics for the participant.

The SOFA score is to be collected baseline at day 3 and day 7, however, date may not be available on these particular days hence this score would be missing. In this situation, we’ll use the value from the day before. If this is not available, then we’ll use the value of the day after. If neither are available, then the value for that particular day will be missing.

**Additional analyses**

The primary analysis looks at the total duration of antibiotic days from randomisation to day 28. However, it is also of interest to assess the duration of antibiotics during the intervention phase only i.e., when the biomarker guidance is in use, and also from randomisation to ICU discharge. Therefore, additional analyses will estimate the treatment effect for the duration of antibiotic days during the intervention phase only and also to the point of ICU discharge. Similarly, we will also estimate the same treatment effects for the daily defined dose (DDD) secondary outcome.

The impact of COVID-19 pandemic on the trial will be assessed on completion of the trial. The baseline demographics and primary outcomes for participants recruited before COVID-19 will be compared to those recruited during COVID-19. The objective is to assess if there is any change in the population considered for the trial. In addition, for participants recruited during the COVID-19 pandemic period, baseline demographics and primary outcomes will be compared for COVID positive versus COVID negative participants. We also perform a sensitivity analysis by dropping COVID positive participants to see if COVID pandemic has an impact on the trial. We will do a sensitivity analysis to account for the non-compliant sites with PCT testing during intervention and follow-up phase. The plan is to run the primary outcome analysis excluding the non-compliant sites and see if any difference between the arms.

**Safety**

The frequency and percentages of serious adverse event and adverse event in the trial will be compared between the treatment arms using wither chi-square test or Fisher’s exact test for which the p-values will be reported. The event type, severity assessment, expectedness and relatedness to intervention will also be summarised by treatment arm.

**Statistical software**

The statistical analyses will be conducted in Stata SE version 17.0.

**Health economic analysis plan**

Decision-analysis modelling will be used to estimate for cohorts receiving each treatment protocol and standard care, the expected costs incurred and patient outcomes; patient outcomes will be evaluated in terms of quality-adjusted life years (QALYs). From these results, incremental cost per QALY ratios will be estimated for the two treatment protocols and standard care. These cost per QALY ratios will be used to form an efficiency frontier which will indicate the most cost-effective strategy at chosen cost per QALY thresholds. Results will be presented at two time periods: firstly, at the end of the study, as directed by the funder’s brief, and secondly using a mathematical model employing a time horizon of a patient’s lifetime, in order that the long-term consequences of avoided mortality or morbidity are appropriately captured.

The structure and methodology used within the mathematical model will be determined when the data are available in order to select the most appropriate approach. The model will be populated with trial data and will include key outcomes such as: rates of mortality; costs associated with length of stay for the index hospitalisation, including escalation of care; costs associated with readmission; and costs associated with antibiotic use. Extrapolations will be made based on the outcomes for each arm at the end of the study resulting in estimates of absolute costs and QALYs for each cohort conditional on the proportion of patients who are alive, and of these, the proportion that would have reduced health-related quality of life in the future. The modelling undertaken will be fully compliant with the current recommendations provided by the National Institute for Health and Care Excellence related to the methods of technology appraisal and will include any changes to this guidance before completion of the work. Extensive sensitivity analyses will be conducted in order to assess the robustness of the results to changes in key assumptions and parameter values.

**References**

1. Rhodes A, Evans LE, Alhazzani W, et al. Surviving Sepsis Campaign: International Guidelines for Management of Sepsis and Septic Shock: 2016. Intensive Care Med. 2017; 43: 304-77.
2. NHS England. Commissioning for Quality and Innovation (CQUIN). Guidance for 2015/16.
3. National Institute for Health and Care Excellence. Antimicrobial stewardship: systems and processes for effective antimicrobial medicine use (NG15). NICE Guidance, 2015.
4. Albrich WC and Harbarth S. Pros and cons of using biomarkers versus clinical decisions in start and stop decisions for antibiotics in the critical care setting. Intensive Care Med. 2015; 41:1739-51.
5. Rhodes A, Evans LE, Alhazzani W, et al. Surviving Sepsis Campaign: International Guidelines for Management of Sepsis and Septic Shock: 2016. Intensive Care Med. 2017; 43: 304-77.
6. Westwood ME, Ramaekers BLT, Whiting P, et al. Procalcitonin testing to guide antibiotic therapy for the treatment of sepsis in intensive care settings and for suspected bacterial infection in emergency department settings: a systematic review and cost-effectiveness analysis. Health Technol Assess. 2015; 19(96).
7. Piaggio G, Elbourne DR, Pocock SJ, Evans SJW, Altman DG, for the CONSORT Group. Reporting of noninferiority and equivalence randomized trials. Extension of the CONSORT 2010 statement. JAMA. 2012; 308(24): 2594-2604.
8. Pocock SJ, Ariti CA, Collier TJ, Wang D. The win ratio: a new approach to the analysis of composite endpoints in clinical trials based on clinical priorities. Eur Heart J 2012 Jan;33(2):176-82.
9. Felker GM, Maisel AS. A global rank end point for clinical trials in acute heart failure. Circ Heart Fail 2010 Sep;3(5):643-6.
10. Armstrong PW, Westerhout CM, Van de Werf F, Califf RM, Welsh RC, Wilcox RG, et al. Refining clinical trial composite outcomes: an application to the Assessment of the Safety and Efficacy of a New Thrombolytic-3 (ASSENT-3) trial. Am Heart J 2011 May;161(5):848-54.
